# Antioxidant Activity and Total Phenolic Content of *Nerium oleander* L. Grown in North of Iran 

**Published:** 2012

**Authors:** Maryam Mohadjerani

**Affiliations:** *Department of Molecular and Cell Biology, Faculty of Basic Science, University of Mazandaran, Babolsar, Iran. *

**Keywords:** Nerium oleander, Total phenolic content, Reducing power, DPPH scavenging activity, Antioxidant activity

## Abstract

In this study, we have examined the antioxidant activity and total phenolic content of different extracts (including water, methanol, water : methanol and acetone) of *Nerium oleander *L. grown in the north of Iran by employing various *in-vitro *assay, *i.e. *DPPH free radical scavenging, reducing power and total antioxidant capacity through the Mo (VI) reduction. The extracts showed different levels of efficacy in each assay in a dose-dependent manner. Methanolic and aqueous methanolic extracts with the highest amount of total phenolic (by using the Folin-Ciocalteu phenol reagent method), were the most potent antioxidant in all of the assays that were used. According to the results of present study, *Nerium oleander *L. flowers were found to serve as a potential source of natural antioxidants.

## Introduction

Reactive oxygen species (ROS) in the forms of superoxide anions, hydroxyl radicals and hydrogen peroxide are generated from the auto-oxidation of lipids, as well as reactive nitrogen species (RNS) (1). Formations of these excess ROS and RNS by Ultraviolet (UV) irradiation, smoking and drug metabolisms are likely to damage several cellular components such as lipids, proteins, nucleic acids, and DNAs through the oxidation or nitration processes (2). In addition, these reactive oxygen species cause inflammation or lesions on various organs and are associated with various degenerative diseases, including cancer, ageing, arteriosclerosis, and rheumatism (3). 

All aerobic organisms, including human beings, have antioxidant defences that protect against oxidative damages, numerous damage removal and repair enzymes to remove or repair damaged molecules (4). However, these natural antioxidant mechanisms can be inefficient (5-8). Although some synthetic antioxidant, such as butylated hydroxyanisole (BHA) and butylated hydroxytoluene (BHT) are commonly used in processed foods, they have been reported to have some side effects (9-11). Therefore, recent search to find natural originated antioxidant has been increased. 

*Nerium oleander *L. is an evergreen shrub or small tree in the dogbane family *Apocynaceae *and is planted throughout the tropical region of the world as garden and roadside trees. Previously cardiotonic, antibacterial, anticancer and anti platelet aggregation activities and also its depression in the central nervous system have been reported from these species (12, 13). Flavonoids are a class of compounds isolated in large quantities from genus of *Nerium*. Flavonoids account for approximately two thirds of the phenolics in our diet as well as a high percentage of the secondary metabolites in *Nerium oleander *(14). There is also a report on antioxidant activity of essential oil of *Nerium oleander *flower (15).

However, owing to the differing antioxidant potential of compounds with different polarities in complex samples, all methods for assessing the antioxidant capacity of these samples are strongly affected by solvents extraction. The purpose of this study was to determine the total phenolic content and the antioxidant activity of water, methanol, water : methanol and acetone extracts of the leaves and flowers of *Nerium oleander *by employing various *in-vitro *methods including the reducing power, total antioxidant capacity and 2,2-diphenyl-1-picrylhydrazyl (DPPH) radical scavenging activity.

## Experimental

*Chemicals*

All chemicals and solvents used were of analytical grade. Folin-Ciocalteu reagent, 1,1-diphenyl-2-picrylhydrazyl (DPPH), potassium ferricyanide, trichloroacetic acid, ferric chloride, Butylated hydroxyanisole (BHA), ascorbic acid, gallic acid, sodium phosphate dibasic, and sulfuric acid were purchased either by Sigma chemical or Merck Co. The aerial parts of *Nerium oleander *were collected from Babolsar in September 2010 and identified by Dr. A. Naghinezhad. A voucher specimen is deposited in the herbarium of the University of Mazandaran (Nr. 1500).

*Extraction procedures*

The extraction from dried and finely powdered leaves and flowers of *Nerium oleander *were carried out using four different solvents to compare the effect of extraction solvents on the total phenolic content, reducing power, radical scavenging and antioxidant activity. These solvents included methanol, methanol : water (50 : 50, v/v), water and acetone. The plant sample (1 g) was extracted with 3 × 20 mL of solvent under stirring at room temperature, till the solvent became colorless. Each extract was centrifuged (3500 rpm; 5 min.) and filtered through Whatman No. 1 filter paper. The filtrates were evaporated to dryness in vacuo at 40°C in a rotavapor. The dried samples of each extract were weighed to determine the yield of soluble constituents and stored at 4°C until use.

*Determination of total phenolic content*

The total phenolic content of each extract was assessed approximately by using the Folin-Ciocalteu phenol reagent method (16). Briefly, 20 μL of each extract solution was mixed with 1.58 mL distilled water and 100 μL of Folin-Ciocalteu reagent, followed by the addition of 300 μL of Na2CO3 solution (7%) after 1 min. Subsequently, the mixture was incubated by shaking at room temperature for 2 h. Then, its absorbance was measured at 760 nm. Gallic acid was used as a standard for calibration curve. The phenolic content was expressed as gallic acid equivalents in milligrams per gram of the dried material using the following linear equation based on the calibration curve:

A = 0.0468 C + 0.0006

R^2 ^= 0.9968

Where A stands for the absorbance and C is the concentration as gallic acid equivalents (μg/mL).

*Reducing power assay*

The reductive potential of the extracts was determined according to the method prescribed by Oyaizu (17). Briefly, the different concentrations of the extracts, the standard compounds, ascorbic acid and gallic acid, (5-100 μg) in 1 mL of distilled water were mixed with 2.5 mL of phosphate buffer (0.2 M, pH = 6.6) and 2.5 mL of potassium ferricyanide [K3Fe (CN)6] (1% w/v). The reaction mixtures were incubated at 50°C for 20 min. A portion (2.5 mL) of trichloroacetic acid (10% w/v) was added to the mixtures. A volume of 2.5 mL of solutions were mixed with 2.5 mL of distilled water and 0.5 mL of FeCl3 (0.1% w/v). The absorbance of all the samples solutions were measured at 700 nm. An increase in the absorbance of the reaction mixture indicated greater reducing power. *DPPH radical scavenging activity*

The ability of the extracts to scavenge the DPPH radicals was determined according to the method of Blois (18). Briefly, 1 mL of methanolic solution of DPPH˙ (0.1 mM) was mixed with 3 mL of extract solution in methanol (containing 2-25 μg of the dried extract). The mixture was then vortexed vigorously and allowed to stand at room temperature for 15 min. The absorbance was measured at 515 nm and its activity was expressed as the percentage of DPPH˙ scavenging relative to the control sample using the following equation:

100 ×DPPH scavenging activiyy (%)=

Absorbance of control - Absorbance of sampleAbsorbance of control

*Total antioxidant capacity*

This assay is based on the reduction of Mo (VI) to Mo (V) by the sample and the subsequent formation of a green phosphate/Mo (V) complex at acidic pH (19). An aliquot of 0.3 mL of the sample solution (containing 100 μg of the dried extract in the corresponding solvent) was combined in a glass tube with 3 mL of reagent solution (0.6 M sulphuric acid, 28 mM sodium phosphate, and 4 mM ammonium molybdate). The tubes were incubated in a boiling water bath at 95°C for 90 min. Then, the samples were cooled to room temperature and the absorbance of the resultant solution was measured at 695 nm against the blank. A typical blank solution was prepared in the same conditions by replacing the sample with an appropriate volume of the same solvent used for the sample. *Statistical analysis*

All the experiments were carried out in triplicates and the results are reported as mean ± standard deviation. The analysis was performed using GraphPad Prism for windows.

## Results and Discussion

*Extract yield and total phenolics*

The yields and amounts of the total phenolic contents for different extracts from the leaves and flowers of *Nerium oleander *are listed in Table 1. 

**Table 1:** Extract yield and total phenolic contents of different solvent extracts from *Nerium oleander*.

**Leaves extracts**	**Yield (%)**	**Total phenolics (%)**	**Flowers extracts**	**Yield (%)**	**Total phenolics (%)**
**LM**	18.7	4.54 ± 0.23	FM	48.8	7.52 ± 0.93
**LMW**	22.1	4.25 ± 0.23	FMW	41.4	7.15 ± 0.43
**LA**	12.6	2.08 ± 0.38	FA	13.7	6.24 ± 0.57
**LW**	11.6	4.21 ± 0.29	FW	35.1	7.13 ± 0.49

All the values are mean ± SD; SD: Standard Deviation. The phenolic contents are expressed as μg gallic acid equivalent per 100 μg extract. L: Leaves; F: Flowers; M: Methanol; W: water; MW, Methanol-Water; A: Acetone.

The amount of the total phenolic content expressed as mg gallic acid equivalent per g of dried material ranged from 2.628 in the acetone extract to 9.402 in the aqueous methanol extract and 8.547 in the acetone extract to 36.690 in the methanol extract for the leaves and flowers respectively. Aqueous methanol and methanol were found to be the most effective solvents in the extraction of the phenolic content from *Nerium oleander *leaves and flowers, respectively. This is in agreement with the reports in literature that methanol is a widely used and effective solvent for extraction of phenols.

*Reducing power*

**Figure 1:**
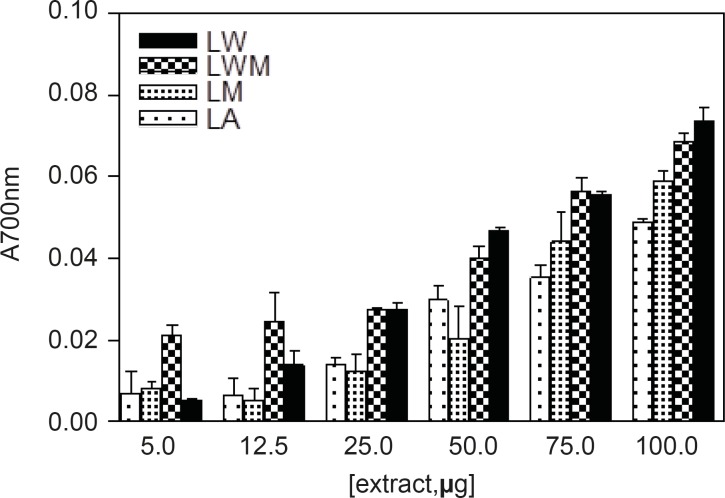
Reducing power of leaves of *Nerium oleander *indicating the amount of dried extract added into the test sample. The standard ascorbic acid and gallic acid were used. For abbreviation refer to Table 1

**Figure 2:**
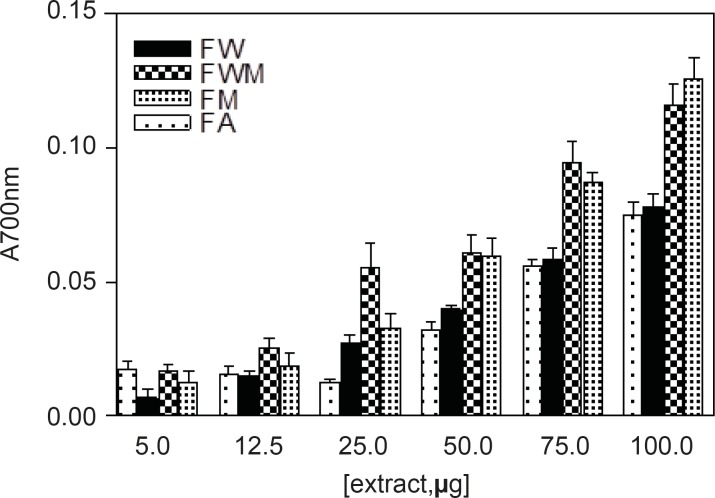
Reducing power of flowers of *Nerium oleander *indicating the amount of dried extract added into the test sample. The standard ascorbic acid and gallic acid were used. For abbreviation refer to Table 1

Different studies have indicated that the reducing capacity of bioactive compounds is associated with the antioxidant activity (20). In this study, the reducing power of *Nerium oleander *extracts and the reference compounds (ascorbic acid and gallic acid) were determined. All extracts showed some degree of electron donation capacity in a concentration-dependent manner (Figures 1 and 2), but the capacities were lower than that of ascorbic acid or gallic acid. The methanolic and aqueous methanolic extracts of the leaves containing the highest amount of total phenolics, were the most potent reducing agents, whereas the acetone extract containing that showed the least amount of phenolics content, was the weakest in the activity.

*Reducing power*

Different studies have indicated that the reducing capacity of bioactive compounds is associated with the antioxidant activity (20). In this study, the reducing power of *Nerium oleander *extracts and the reference compounds (ascorbic acid and gallic acid) were determined. All extracts showed some degree of electron donation capacity in a concentration-dependent manner (Figures 1 and 2), but the capacities were lower than that of ascorbic acid or gallic acid. The methanolic and aqueous methanolic extracts of the leaves containing the highest amount of total phenolics, were the most potent reducing agents, whereas the acetone extract containing that showed the least amount of total phenolics, were the most potent reducing agents, whereas the acetone extract containing that showed the least amount of phenolics content, was the weakest in the activity.

*Reducing power*

Different studies have indicated that the reducing capacity of bioactive compounds is associated with the antioxidant activity (20). In this study, the reducing power of *Nerium oleander *extracts and the reference compounds (ascorbic acid and gallic acid) were determined. All extracts showed some degree of electron donation capacity in a concentration-dependent manner (Figures 1 and 2), but the capacities were lower than that of ascorbic acid or gallic acid. The methanolic and aqueous methanolic extracts of the leaves containing the highest amount of total phenolics, were the most potent reducing agents, whereas the acetone extract containing that showed the least amount of phenolics content, was the weakest in the activity.

*DPPH radical scavenging activity*

**Figure 3:**
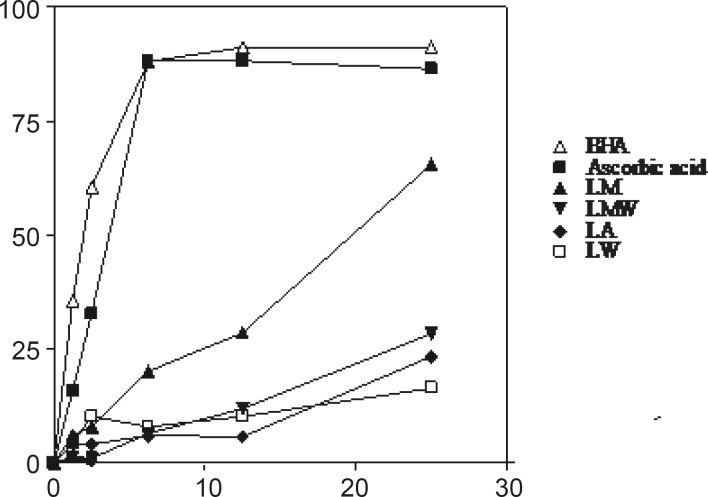
DPPH radical scavenging activity of four extracts of *Nerium oleander *leaves. BHA and ascorbic acid were used as positive controls. Each point was averaged from triplicate measurements with standard deviations falling within the data points. For abbreviation refer to Table 1

**Figure 4:**
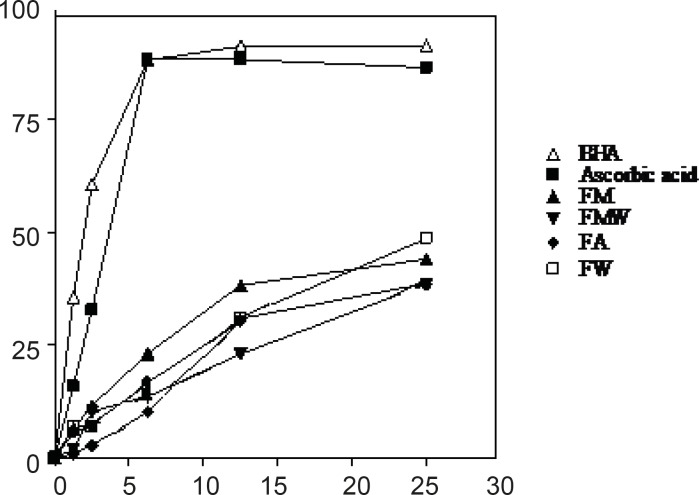
DPPH radical scavenging activity of four extracts of *Nerium oleander *flowers. BHA and ascorbic acid were used as positive controls. Each point was averaged from triplicate measurements with standard deviations falling within the data points. For abbreviation refer to Table 1.

The DPPH radical has been extensively used to evaluate the reducing substances (21) and is a useful reagent for investigating the free radical scavenging activities of compounds. The radical scavenging activity of four extracts from *Nerium oleander *leaves and flowers, ascorbic acid and BHA at five concentrations was tested by DPPH method and the results are illustrated in Figures 3 and 4. The methanolic extracts from the leaves and flowers that contained the highest amounts of the total phenolics, were found to be the most active radical scavengers followed by aqueous methanol and acetone extracts. Interestingly, the extracts from the leaves showed relatively high DPPH scavenging activity comparing with those extracts from the flowers of *Nerium oleander*. 

**Table 2:** Comparison of reducing power of leaves and flowers in different solvents

**Leaves extracts**	**Reducing Power (100 μg extract in test)**	**Flowers extracts**	**Reducing Power (100 μg extract in test)**
**LM**	0.059 ± 0.004	FM	0.126 ± 0.014
**LMW**	0.068 ± 0.004	FMW	0.116 ± 0.013
**LA**	0.049 ± 0.001	FA	0.075± 0.008
**LW**	0.074 ± 0.005	FW	0.078 ± 0.008

All the values are mean ± SD; SD: standard deviation. There was 100 *μ*g of indicated extract in each sample.

Standard ascorbic acid and gallic acid were used and absorbances in 700 nm were 0.795 ± 0.015 and 1.913 ± 0.090, respectively. For abbreviation refer to Table 1.

*Total antioxidant capacity*

Using the phosphomolybdenum assay, which is a quantitative method to evaluate the water-soluble and fat-soluble antioxidant capacity (total antioxidant capacity), shows the four extracts exhibited different levels of activity in a concentration-dependent manner. A higher activity was observed for 100 μg extract in the methanolic leaves extract (1.281 mg of ascorbic acid equivalents/mg extract) and in the aqueous extract of the flowers (2.930 mg of ascorbic acid equivalents/mg extract) (Table 3).

**Table 3:** Comparison of the total antioxidant activity of four extracts of *Nerium oleander *and BHA..

**Sample**	**Antioxidant activity**a†	**Sample**	**Antioxidant activity**
**LM**	1.280 ± 0.02	FM	2.330 ± 0.04
**LMW**	1.246 ± 0.01	FMW	1.386 ± 0.02
**LA**	0.982 ± 0.01	FA	1.596 ± 0.04
**LW**	0.912 ± 0.004	FW	2.930 ± 0.01
**BHA**	6.474 ± 0.05	BHA	6.474 ± 0.05

a: Total antioxidant capacity (phosphomolybdenum assay) was expressed in mg ascorbic acid equivalents/mg extract); †: 100 μg of dried extracts and BHA were used; All the values are mean ± SD; SD: standard deviation, (n = 3); For abbreviation refer to Table 1.

In conclusion, we showed that *Nerium oleander *possesses an effective antioxidant activity, which includes free radical scavenging and reducing power. Various solvent extracts from *Nerium oleander *leaves and flowers showed different levels of antioxidant activity in different test systems in a concentration- dependent manner. The antioxidant activity was correlated with the amount of the total phenolic content present in the respective extracts in each assay. Methanol and aqueous methanol were proved to be the most efficient solvents for the extraction of antioxidants from *Nerium oleander *flowers and leaves. The related extract contains the highest amount of phenolic contents and also exhibited the strongest antioxidant capacity in all the assays used here. These findings are useful for further research to identify, isolate and characterize the specific compound which is responsible for the higher antioxidant activity.
